# Editorial: Friend or foe? - The role of macrophages and neutrophils in liver pathologies

**DOI:** 10.3389/fimmu.2026.1944204

**Published:** 2026-08-06

**Authors:** Mirja Hommel, Lara Campana, Nico Lachmann

**Affiliations:** 1 Gene Therapy for Rare Diseases, Centre for Applied Medical Research, Pamplona, Spain; 2 Resolution Therapeutics, Edinburgh, United Kingdom; 3 Institute for Experimental Hematology, Medizinische Hochschule Hannover, Hannover, Germany

**Keywords:** disease, dual function, liver, macrophages, neutrophils, repair

In recent years our understanding of both macrophages and neutrophils in tissue damage and regeneration has been greatly challenged. Once considered to be pro-inflammatory first responders to pathogens, they were found to possess both pro- and anti-inflammatory functions – a still rather simplistic view that in the past decade has been constantly reviewed. Recent research has uncovered their prominent role in tissue regeneration, especially in the context of liver disease. This special Research Topic combines several primary research and review articles shedding light on how current paradigms are rapidly evolving in the field.

The role of macrophages and neutrophils in liver disease is determined by multiple factors, amongst which localization is probably one of the most important ones. To set the stage, Kim and Lachmann discuss neutrophil and macrophage zonation in liver disease, highlighting how the liver’s portal-central architecture creates spatially graded immune niches characterized by differences in oxygen tension, nutrient flux, antigen exposure, stromal signals and hepatocyte metabolism. Rather than representing a static anatomical feature, hepatic immune zonation is a dynamic and disease-dependent process in which immune-cell positioning, functional states and reprogramming are continuously reshaped by the evolving tissue microenvironment during metabolic stress, tissue injury and infection. Neutrophils, for example, may preferentially accumulate in necrotic or pericentral injury zones, whereas macrophage subsets acquire region-specific identities that contribute differentially to inflammation, fibrosis, tissue repair and host defense. The review further emphasizes that single-cell and spatial omics, intravital imaging, multiplexed histology and AI-supported computational analysis are now redefining hepatic immune microanatomy and may support precise disease stratification and therapeutic targeting.

Focusing on macrophages, three reviews provide a deeper understanding of their heterogeneity, plasticity and potential therapeutic strategies, as well as the challenges the field still faces. Chen et al. compare acute versus chronic settings, which are characterized by a sequential (acute) versus simultaneous (chronic) existence of friends (resolving) and foes (pro-inflammatory). They give an overview of the different subpopulations in various diseases and briefly describe how each shapes disease progression. Wang et al. expand on the mechanisms that lead to macrophage heterogeneity and plasticity on a more molecular level and describe the role of macrophages in chronic liver disease of distinct etiologies that progress to liver fibrosis and beyond.

González del Barrio et al. go more into the details of macrophage heterogeneity in the context of MASLD/MASH. A hallmark of MASLD/MASH is the loss of Kupffer cells and their replacement by TREM2-expressing monocyte-derived macrophages, also referred to as lipid-associated macrophages, or LAMs. The authors highlight that the exact nature of LAMs – like other macrophage populations – is far from being black-and-white and that the development of novel tools and techniques is required to elucidate how they develop, the full extent of their functions and how to potentially target them. Importantly, this review highlights the limitations of the now-deprecated M1/M2 dichotomy and adopts a more accurate framework in which macrophages are viewed as existing along a phenotypic spectrum — a framework more likely to facilitate the identification of novel therapeutic interventions.

All three reviews discuss current macrophage-targeted or -based therapeutic approaches. Initially, the field was primarily focused on immunosuppression, but this view has been replaced by a need to immune-modulate the chronically damaged liver to promote tissue remodeling and regeneration. They narrow down on the need to adopt precision medicine approaches, ranging from pharmacological modification of and nanodrugs aimed at specific subpopulations to cell therapies, including adoptive transfer of macrophages with a pro-regenerative phenotype, chimeric antigen-receptor macrophages (CAR-Ms) and signal-converting CAR-Ms. Another strategy being explored is the specific elimination of disease-associated subpopulations. However, while some advances have been made, especially with clinical-stage research into regenerative macrophages, further investigation is needed to turn promising clinical and preclinical data into approved therapies.

In both MASLD and ALD, the liver-gut axis is implicated in the progression to fibrosis, cirrhosis and HCC. Two reviews in our Research Topic address the role of macrophages and neutrophils in the physiology of the epithelial, vascular, and hepatic immune barriers along this axis, and their deterioration in pathological conditions. Aslan et al. highlight how distinct macrophage populations, each with a specific ontogeny and niche, act as gatekeepers of barrier integrity under physiological conditions, thereby preventing bacterial translocation. In cirrhosis, the failure of such barrier in fact drives an increase in bacterial translocation, which is associated with a significant risk of infection. Yin et al. provide a new insight into the role of neutrophil extracellular traps (NETs) in the onset and progression of MASLD: NETs directly promote an inflammatory response in the liver whilst also facilitating the transfer of bacterial products to the liver, thereby creating a dual-hit situation that promotes disease progression. Thus, when designing therapeutic approaches, modulating the gut microbiome may be beneficial to the improvement of inflammation.

In a piece of original research, Gao et al. investigated core genomic signatures of NETs in the context of ASH. Using patient samples, they identified a three-molecule signature, FOS, MMP7, and CXCL6, which allows to distinguish two subtypes of ASH, namely a metabolic-dominant and a pro-inflammatory one. If confirmed, this could improve our ability to stratify ASH patients for prognostic and/or drug development purposes. Furthermore, using drug-gene interaction analysis, they identified new modulators of this signature, including N-acetylcysteine, which may pave the way for a future therapeutic intervention.

Lastly, in a systematic review Pan et al. elucidated the usefulness of mesenchymal stem cell-derived small extracellular vesicles (sEVs) as therapeutic tool for sepsis-related injury in rodent models.

Taken together, the articles in this Research Topic highlight the significant heterogeneity and plasticity of liver-associated macrophage populations and the challenges but also the opportunities that this variability brings for a successful development and application of therapeutic approaches. They also emphasize the role of neutrophils in certain liver pathologies and remind that it is important not to see the liver as an isolated organ. Finally, they highlight a fundamental concept: our ability to productively manipulate non-parenchymal cell biology in liver disease may be the key to future disease-modifying therapeutic interventions, especially in advanced or decompensated cirrhosis, where the microenvironment is entirely subverted by repeated cycles of damage and remodeling.

